# Renal manifestations of HIV during the antiretroviral era in South Africa: a systematic scoping review

**DOI:** 10.1186/s13643-017-0605-5

**Published:** 2017-10-13

**Authors:** Shirelle Assaram, Nombulelo P. Magula, Suman Mewa Kinoo, Tivani P. Mashamba-Thompson

**Affiliations:** 1 Department of Internal Medicine, Nelson R. Mandela School of Medicine, University of KwaZulu-Natal, 719 Umbilo Road, Congella, Durban, 4013 South Africa; 2 Department of General Surgery, Nelson R. Mandela School of Medicine, University of KwaZulu-Natal, Durban, South Africa; 3 Department of Public Health Medicine, School of Nursing and Public Health, University of KwaZulu-Natal, Durban, South Africa

**Keywords:** HIV, Antiretroviral treatment, Renal failure, South Africa

## Abstract

**Background:**

It is estimated that 650,000 patients may develop human immunodeficiency virus (HIV)-related renal disease in South Africa. South Africa has recently adopted WHO policy, stipulating that all HIV-infected patients have access to antiretroviral treatment (ART) irrespective of CD4 cell count.

**Methods:**

We searched Google Scholar, PubMed, Medline, Cochrane Library, Worldcat.org and EBSCO host databases from July 2015 to December 2015. Eligibility criteria included articles pertaining to renal manifestations of HIV in South Africa from 2004 to 2015 in adult patients (≥ 18 years). We independently reviewed the articles for quality. Thematic content analysis was performed to identify patterns of renal manifestations from the included studies. The risk of bias (e.g. internal validity) in the included studies was evaluated using the mixed methods appraisal tool.

**Results:**

Eleven out 21 studies were eligible for data extraction. The prevalence of urine abnormalities on urine dipsticks was high but had poor sensitivity and specificity for detecting renal impairment. Normal renal function occurred in 28.4 to 79% of patients, mild renal impairment occurred in 19 to 57.1% and moderate renal impairment in 2 to 14.4%. Severe renal impairment occurred in 1.3% of patients. Both the Cockcroft-Gault equation (after correcting for bias) and the 4-variable Modification of Diet in Renal Disease equation (without the ethnicity factor for African Americans) have been validated for the estimation of glomerular filtration rate (eGFR) in Black South Africans. HIV-associated nephropathy was the most prevalent histology seen (57.2%). Older age, a lower CD4 count, a low haemoglobin and a detectable viral load were associated with renal impairment. Renal function improved in the first year of commencing ART as evidenced by the regression of proteinuria and the increase in eGFR.

**Conclusion:**

The findings of the review have implications to the recently adopted ‘test and treat’ approach to HIV prevention and management.

**Systematic review registration:**

PROSPERO CRD42016039270

**Electronic supplementary material:**

The online version of this article (10.1186/s13643-017-0605-5) contains supplementary material, which is available to authorized users.

## Background

In April 2004, the South African National antiretroviral treatment (ART) guidelines were first implemented [1, 2] and ART became universally accessible in the public sector for the first time. South Africa recently (01 September 2016) adopted the World Health Organization (WHO) “test and treat” approach, which was introduced as a possible means of controlling the global HIV epidemic [3]. This approach entitles every patient who tests positive for HIV to a lifelong ART regardless of their CD4 count or clinical staging [3]. With the successful rollout of ART in South Africa, the lifespan of people living with human immunodeficiency virus (HIV) has been prolonged thereby transforming HIV into a disease of chronicity [4], adding to the burden of infectious and non-communicable diseases [5]. Renal disease is a recognised complication of HIV infection and its incidence can be perpetuated by drug induced toxicity, comorbid diseases such as diabetes and hypertension and infectious diseases [6]. Data from the USA suggest that at some stage of their HIV infection, 10% of patients will develop HIV-related renal disease [7]. If this is extrapolated to the South African context, it is estimated that 650,000 patients may develop HIV-related renal disease [7]. This large burden of chronic kidney disease (CKD) would place immense pressure on our resource-strained health system where access to renal biopsy, renal replacement therapies and nephrologists is limited [8].

Mayosi et al. report a 67% increase in deaths related to nephritis/nephrosis in South Africa from 1999 to 2006 causally linked to the increasing HIV prevalence [5]. In their systematic analysis, Stanifer et al. found that CKD is a prevalent and potentially growing disease in Sub-Saharan Africa with 24% of hypertensives, 18.9% of diabetics and 10% of HIV-infected patients having co-morbid CKD [9]. The guidelines published by the Infectious Diseases Society of America (IDSA) in 2014 recommend that all individuals be assessed for kidney disease at the time of HIV diagnosis by way of a screening urinalysis for proteinuria and a calculated estimate of renal function in order to detect renal disease early [10]. The South African ART guidelines incorporate this policy, and the recent implementation of ART initiation irrespective of CD4 allows for earlier access to ART before the onset of advanced disease [3].

Despite the earlier initiation of ART and the screening for urinary abnormalities, renal disease in HIV infected patients is still prevalent [11]. In addition, HIV-associated nephropathy (HIVAN) has become the third leading cause of end stage renal disease among HIV infected African-American patients [12, 13]. To the best of our knowledge, this is the first systematic review that attempts to map the evidence of renal disease in people infected with HIV in South Africa post 2003 when ART became universally accessible to patients in the public sector. In light of the current upsurge of research and publications on the topic [12, 14–17], the contribution of a systematic scoping review gains importance and relevance by demonstrating the current data in order to identify research gaps and suggest novel ideas for future research.

## Methods

### Study design

The protocol of this study is registered in PROSPERO with registration number: CRD42016039270 and available via this website: http://www.crd.york.ac.uk/PROSPERO/display_record.asp?ID=CRD42016039270.

In this study, we chose to undertake a systematic scoping review of published reviews as the best method to map the renal manifestations of HIV in South Africa during the ART rollout period from 2004 onwards. Guided by Arksey and O’Malley’s scoping review framework [18], we searched cross-sectional studies, randomised controlled trials, non-randomised controlled trials, observational studies, review articles, case reports and systematic reviews that examined renal manifestations on HIV-infected patients in South Africa.

### Literature search

We conducted a systematic literature search in the following databases: Google Scholar, PubMed, Medline, Cochrane Library, Worldcat.org and EBSCO host, for articles pertaining to renal manifestations of HIV in South Africa. The database search occurred from July 2015 to December 2015. The primary search terms related to HIV and the kidney specifically (i.e. HIV, kidney, renal, nephrology). The secondary terms were manifestations, renal failure, complications, South Africa, antiretroviral treatment, proteinuria and glomerular filtration rate (for a detailed description of the database search strategy, see Additional file 1). In order to direct the search to our research question, we used the filtering method which included the data range (2004 to 2015), human subjects, English language and adult patients (≥ 18 years old). Medical subject headings (MeSH) terms were also used.

### Eligibility criteria

#### Inclusion criteria

- Evidence of renal manifestations in HIV infected patients in South Africa
- Evidence from the period 2004 to 2015 (ART rollout began in April 2004 in SA)
- Evidence of renal manifestations in adult HIV infected patients (≥ 18 years old)
- English language publications
- Studies included irrespective of ART status, i.e. on treatment or not on treatment

#### Exclusion criteria

- Evidence of renal manifestations in HIV infected patients outside South Africa
- Studies reporting exclusively on other HIV manifestations
- Studies before the ART era (prior to 2004 ART was not accessible in the public sector)
- Non English language studies

**Fig. 1:**
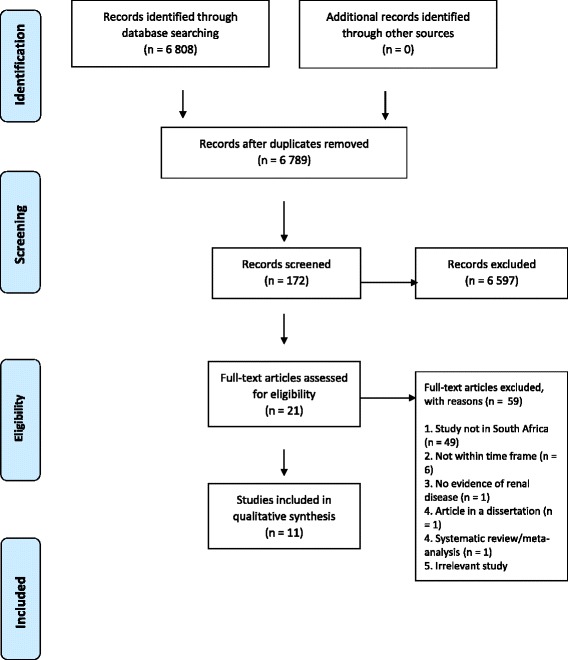
Literature Search and Selection of Studies

Study selection occurred in two stages. First, a single reviewer went through the titles from the database search and decided on eligibility based on the inclusion and exclusion criteria. For example, titles stating research carried out in an ineligible country would be excluded. If a reviewer was uncertain of the eligibility of a title, it was not excluded but rather carried onto the next stage of the selection process. In the second stage, two independent reviews of the titles and abstracts, using inclusion and exclusion criteria, was undertaken. Discrepancies were resolved by discussion until consensus was reached. The remaining articles were then assessed for eligibility for data extraction. A PRISMA flow diagram (Fig. 1: Literature Search and Selection of Studies) shows the process involved in obtaining eligible studies. The PRISMA checklist is provided as an Additional file 2.

### Quality of the evidence

The risk of bias (overall quality of each article) in the included studies was evaluated using the mixed methods appraisal tool (MMAT) for mixed methods studies [19] (Additional file 3). The risk of bias scale scored studies was based on 14 criteria. Quantitative studies were assessed by the following domains: the clarity of the research questions, confidence in the assessment of the research question, appropriateness of data sources collected, suitability of statistical analysis to address the research question, confidence in the assessment of exposure, acknowledgement of possible researcher bias, appropriateness of the sampling strategy, representativeness of population, confidence in the measurements outcome and an acceptable response rate. The non-randomised study was assessed by the following domains: minimization of selection bias during recruitment, confidence in the assessment of exposure, matching exposed and unexposed variables for all those that are associated with the outcome of interest or the adjustment of statistical analysis for these prognostic factors, confidence in the assessment of outcome or adequacy of the follow-up of cohorts. For each included study, an overall percentage quality score was calculated. The scores are presented using descriptors such as *(≤ 25%)-poor, **(26–50%)-fair, ***(51–75%)-average, and ****(76–100%)-good (Additional file 3). Two reviewers (SA and SMK) independently performed each quality assessment. Differences in ratings were resolved through discussion.

### Thematic analysis

Thematic content analysis was performed to identify patterns of renal manifestations from the included studies. The included manuscripts were manually coded into categories which were grouped into the following five themes:

- Urine analysis
- Estimated glomerular filtration rate
- Renal biopsy
- Risk factors for renal dysfunction
- Clinical and histological responses to ART

## Results

A total of 6808 articles were retained from our initial search. Applying our exclusion criteria reduced the number of studies to 11 (Figure 1). Level of agreement between reviewers was 80 versus 50% expected by chance. This constitutes moderate to substantial agreement, with estimated kappa statistic = 0.22 95% (confidence interval [CI] 0.70; 0.26).

A total of 6758 studies were excluded as they did not meet the inclusion criteria for this study. Of those, 10 underwent a full manuscript review and were found to have no valuable data for analysis in this study for the following reasons: not within the specified time frame [12, 17, 20–23], outcomes of chronic haemodialysis [24], systematic review and meta-analyses of countries in Sub-Saharan Africa (9), irrelevant study population [25], and a dissertation published at a later stage [26].

### Characteristics of included studies

Eleven out of the 21 reviewed articles were eligible for data extraction (Additional file 4). Of these, eight were conducted in an urban setting [15, 16, 27–32] and three were carried out in a rural setting [33–35]. Rural is defined as sparsely populated areas in which people farm or depend on natural resources, including the villages and small towns that are dispersed through these areas. In addition, in South Africa, they include the large settlements in the former homelands, created by the apartheid removals, which depend for their survival on migratory labour and remittance. All included studies were published between 2008 and 2015. Study participants were predominantly female (total female percentage 57.9%) in all included studies except two [29, 30]. The total sample size of all 11 studies was 6595 participants. The included number of participants in each study was 100 participants or more. The average age in all the included studies was 38.6 ± 4.5 years. The study designs of the included studies were as follows: two retrospective cohort studies [27, 29], one cohort study [31], two prospective cohort studies [32, 33], one non-randomised control study [15], one case control study [28], and four cross-sectional studies [16, 30, 34, 35].

All the included studies were aimed at assessing the renal status, risk factors and renal outcomes in HIV-infected patients either before ART, whilst on ART or both. Seven of the 11 studies (63.6%) assessed for the prevalence of renal impairment in HIV-infected patients [16, 27, 29, 32, 33, 35], two studies (18.1%) analysed renal biopsies to evaluate the clinical and histological responses of HIV-associated kidney disease to ART [15, 31], one study (9%) assessed the performance of the 4-variable Modification of Diet in Renal Disease (4-v MDRD) and Cockcroft-Gault (CG) equations for estimating glomerular filtration rate (eGFR)/creatinine clearance in South African Black patients [30], and one study (9%) extracted genomic DNA from kidney samples to determine the prevalence of APOL1 risk variants and their effect on CKD in Black South Africans [28]. General and specific characteristics of all included studies are described in Additional file 4. The evidence of renal dysfunction in the included studies is summarised in Table 1.

**Table 1:** Evidence of renal dysfunction in the included studies

Author	Markers for renal impairment	Definition of renal disease	Results
Brennan et al. 2011 [27]	Creatinine clearance using Cockcroft-Gault (CG) equation.	Nephrotoxicity defined as any decline in kidney function from baseline (acute or chronic) that is secondary to a toxin (including drugs) documented within 48 months of initiating tenofovir (TDF). Normal renal function (> 90 ml/min), mild renal dysfunction (60–89 ml/min) and moderate renal dysfunction (30–59 ml/min)	The risk of nephrotoxicity and death by 48 months increased with decreasing renal function at initiation of TDF. Patients switched onto TDF had a higher risk of nephrotoxicity and death compared to ART naïve patients. Median time to nephrotoxicity after TDF initiation is 3.6 months confirming the importance of the month 3 creatinine clearance.
Fabian et al. 2009 [16]	Urine dipstick for proteinuria and microalbuminuria	Microalbuminuria: microalbumin-to-creatinine ratio 3.4–33.9 mg/mmol independent of sex; overt proteinuria: protein-to-creatinine ratio of 0.03–0.3 g/mmol; nephritic range proteinuria: protein-to-creatinine ratio > 0.3 g/mmol.	18.5% had microalbuminuria, 6.4% had overt proteinuria and 2.4% had nephrotic range proteinuria.
Fabian et al. 2013 [15]	Urine dipstick for proteinuria. Estimated glomerular filtration rate (eGFR) using both CG and 4 variable Modification of Diet in Renal Disease (MDRD) formulae. Renal biopsy	Persistent microalbuminuria: microalbumin-to-creatinine ratio of 3.4–33.9 mg/mmol; Persistent overt proteinuria: protein-to-creatinine ratio of 34 mg/mmol-0.3 g/mmol; Nephrotic proteinuria: protein-to-creatinine ratio > 0.3 g/mmol.	68% had microalbuminuria, 23% had overt proteinuria and 9% had nephrotic proteinuria. There was an improvement in eGFR on antiretroviral treatment (ART). There was partial or complete remission of proteinuria in response to treatment. Despite the rapid clinical response to ART, there was relative lack of histological resolution.
Franey et al. 2009 [33]	Urine dipstick for proteinuria. eGFR using the four variable MDRD equation. Urine dipstick for proteinuria	Severe renal impairment: eGFR < 30 mls/min/1.73m^2^; Moderate renal impairment: eGFR 30–60 mls/min/1.73^2^; mild renal impairment: eGFR 60–90 mls/min/1.73^2^. Proteinuria ≥ 1+ protein on dipstick. Renal dysfunction defined as either reduced eGFR and/or proteinuria/haematuria.	Severe renal impairment was uncommon while moderate and mild renal impairment were more common. Mild and moderate renal impairment improve on ART. Urine analysis may not be sufficiently sensitive to be used as a single screening test for renal disease at baseline.
Kamkuemah et al. 2015 [32]	eGFR calculated using CG equation	Severe renal function reduction was defined as eGFR < 30 ml/min/1.73 m^2^, moderate reduction as eGFR of 30–59 ml/min/1.73 m^2^ and mild reduction as an eGFR of 60–89 ml/min/1.73 m^2^.	79% had normal renal function at baseline, 19% had mildly reduced renal function and 2% had moderate renal impairment at baseline. Overall renal function improved over the first year after starting TDF-containing ART regimens.
Madala et al. 2014 [34]	Urine dipstick for proteinuria. eGFR calculated using the MDRD equation for ≥ 18 years and the Schwartz equation for < 18 years old.	Chronic kidney disease (CKD) defined by eGFR < 60 ml/min/1.73m^2^ and/or proteinuria and/or abnormal renal ultrasound, persistent for ≥ 3 months.	eGFR was < 30 ml/min/1.73m^2^ in 50.6% of patients as this was a CKD clinic. Main risk factors for CKD were diabetes, hypertension and HIV.
Vachiat et al. 2013 [29]	Urine for proteinuria either dipstick or spot urine protein creatinine ratio. Serum creatinine levels.	Acute kidney injury (AKI) defined as an improvement in admission serum creatinine of > 50%. They were further subdivided using the rifle criteria: risk—serum creatinine < 194 μmol/L; injury—serum creatinine 195 to 291 μmol/L; and failure—serum creatinine > 291 μmol/L.	Majority had AKI 56%, followed by CKD 23% and 21% had acute on chronic kidney disease. Proteinuria did not predict recovery or death in HIV-infected patients with AKI. AKI was common in HIV-infected patients and occurred at a younger age than HIV negative patients.
Wearne et al. 2012 [31]	Renal biopsy Serum creatinine Urine protein creatinine ratio	HIV-associated nephropathy (HIVAN) defined as a constellation of glomerular, interstitial and tubular abnormalities, and there must be epithelial cell hyperplasia if only tubular or interstitial disease was present.	For patients with HIVAN, there was an improvement in proteinuria and stabilisation of renal function after commencing ART. Renal biopsy is essential to diagnose renal disease in HIV-infected patients.
Wensink et al. 2015 [35]	Urine for albuminuria eGFR calculated using MDRD and CKD epidemiology collaboration formula	Moderately increased albuminuria: albumin creatinine ratio 30 to 299 mg/g; Severely increased albuminuria: albumin creatinine ratio > 300 mg/g.	Albuminuria occurred in 20% of patients while only 2% had eGFR < 60 ml/min/1.73m^2^. Higher eGFR was significantly linked to lower prevalence of albuminuria. Albuminuria was linked to higher frequency of diabetes, hypertension, high total cholesterol and decreased eGFR.

*CG* Cockcroft-Gault, *TDF* tenofovir disoproxol fumarate, *eGFR* estimated glomerular filtration, *MDRD* Modification of Diet in Renal Disease, *ART* antiretroviral treatment, *CKD* chronic kidney disease, *AKI* acute kidney injury, *HIV* human immunodeficiency virus, *HIVAN* HIV-associated nephropathy

### Risk of bias assessment

The studies’ quality scores ranged from 87.5 to 100% (Additional file 2). Six of the 11 included studies scored the highest quality score of 100% [15, 28, 29, 31–33]. The three studies scoring the lowest had an average score of 87.5% each [30, 34], and the remaining two studies had an average score of 91.5% each [16, 35]. Overall, the body of evidence was considered at minimal risk of bias due to the following: description of randomisation was clear, allocation concealment none, retention percentage in study was acceptable, minimal selection bias and, selection and comparativeness of the control groups was adequate.

### Findings of the study

Kidney disease is a complication of HIV infection and can present as acute kidney injury (AKI) or CKD. Only one study assessed AKI (defined as an improvement in admission serum creatinine of > 50%) in HIV-infected ART naïve patients [29]. Their findings demonstrated that 56% had AKI, 21% had acute on chronic kidney disease and 23% had CKD [29]. The causes of AKI included sepsis, volume depletion, haemodynamic instability, toxins, urological obstruction and miscellaneous causes [29].

### Urine analysis

The incidence of urine abnormalities on urine dipsticks was high: 30 to 57% had leukocyturia [16, 29], 16 to 40% had microscopic haematuria [16, 29, 33] and 20 to 44% had microalbuminuria/proteinuria [15, 16, 33, 35]. Two studies investigated the cause of leukocyturia and detected an infective organism, mainly E. coli, in 29.1% of culture positive cases in one study (ART naïve outpatients) [16] and in 8.9% of culture positive cases in the other study (ART naïve outpatients with renal failure) [29]. Franey et al. performed urine dipstick analysis on 149 patients who were initiating ART in a rural clinic, to assess its utility to detect impaired renal function [33]. They concluded that urine dipsticks analysis alone had poor sensitivity and specificity for detecting impaired renal function (PPV 0.22) [33].

In three studies on ART naïve patients, positive proteinuria on dipsticks was quantified by sending the specimen for a spot protein:creatinine ratio (PCR) [15, 16, 29]. If the dipsticks were negative for protein, they were screened for microalbuminuria which, if positive, were then sent for a spot microalbumin-to-creatinine ratio test (MCR) [15, 16]. In their study, Fabian et al. found that, of the 253 dipsticks positive for proteinuria on ART naïve patients, only 193 were confirmed by the laboratory [16]. Wensink et al. [35] submitted random urine samples directly to the lab for the albumin:creatinine ratio (ACR), bypassing urine dipsticks analysis. Two studies quantified the degree of proteinuria using either the MCR [15] or the ACR [35]. Microalbuminuria (MCR 3.4–33.9 mg/mmol) occurred in 68% of patients, overt proteinuria (MCR 34 mg/mmol–0.3 g/mmol) in 23% and nephrotic proteinuria (MCR > 0.3 g/mmol) in 9% of ART naïve patients [15]. Similar figures were noted in the study that quantified the ACR in a mixed study population predominantly on ART (87% on ART): 20% of patients had moderately increased albuminuria (ACR 30–299 mg/g) and 1% had severely increased albuminuria (ACR > 300 mg/g) [35]. In the same study, a detectable HIV viral load, hypertension, total cholesterol and eGFR were all independently associated with albuminuria [35].

### Estimated glomerular filtration rate (eGFR)

Six studies utilised eGFR as a marker of renal dysfunction [15, 27, 30, 32, 33, 35]. eGFR was calculated using the CG equation [27, 32], the 4-v MDRD equation [33], comparing the CG and 4-v MDRD equations [15, 30] or comparing the 2009 Chronic Kidney Disease Epidemiology Collaboration (CKD-EPI) formula and the 4-v MDRD equation [35]. Both the CG equation (after correcting for bias) and the 4-v MDRD equation (without the ethnicity factor for African Americans) have been validated for the estimation of eGFR in Black South Africans [30].

The eGFR was classified as follows: severe renal impairment eGFR < 30mls/min/1.73 m^2^, moderate renal impairment eGFR 30–59 mls/min/1.73 m^2^, mild renal impairment eGFR 60–89 mls/min/1.73 m^2^ and normal renal function eGFR > 90 mls/min/1.73 m^2^. Using the afore-mentioned classification, baseline (before ART initiation) renal function was documented as follows: normal renal function occurred in 28.4% to 79% of patients, mild renal impairment occurred in 19 to 57.1% and moderate renal impairment in 2 to 14.4% [27, 32, 33]. Baseline severe renal impairment occurred in 1.3% of ART naïve patients in the study by Franey et al. [33]. Wensink et al. had a mixed study population of ART naïve patients and HIV-infected patients on ART and the majority of patients had normal renal function, 2% had moderate renal impairment and 10% had mild renal impairment [35].

Brennan et al. confirmed the importance of the 3-month creatinine clearance by demonstrating that the median time to nephrotoxicity after tenofovir disoproxil fumarate (TDF) initiation was 3.6 months [27]. A study by Kamkuemah et al. showed that early serum creatinine testing at months 1 and 2 after initiation of TDF may not be useful in predicting those at risk for renal dysfunction as it had a low predictive value for predicting change in eGFR after a year on ART [32].

### Renal biopsy

Renal histological findings have been inconsistently classified in patients on ART [31]. HIV-associated nephropathy (HIVAN) was the most prevalent histology seen (57.2%) followed by HIVAN with immune complex glomerulonephritis (ICGN) at 21.8%; without HIVAN or ICGN (12.5%); and ICGN in isolation (8.3%) [31]. However, in another study by Fabian et al., there was a paucity of HIVAN lesions on histology [15].

### Risk factors for renal dysfunction

Two studies were unanimous that abnormal renal function at baseline was associated with older age, World Health Organizaton (WHO) stage III/IV disease and a lower CD4 count [32, 33]. In addition to these variables, a low haemoglobin and a detectable viral load were also associated with abnormal renal function in a mixed cohort of ART naïve patients and patients on ART [27, 32]. In the same study, patients who were switched onto TDF from another regimen had a higher risk of nephrotoxicity and death, than ART naïve patients initiated on TDF [27]. Younger age and advanced immunosuppression were found to be risk factors for AKI [29]. There was no consensus regarding gender as a risk factor for renal dysfunction, as one study found women to be at greater risk [32] whilst another found men to be at greater risk [33]. The APOL1 risk allele found on chromosome 22 occurred in more than 30% of African American individuals with HIVAN [36]. Kasembeli et al. showed that 79% of Black patients with HIVAN carried two copies of APOL1 risk alleles as opposed to 2% in the general population, suggesting that ART naïve Black South Africans were at high risk of developing HIVAN(28). CKD (defined as eGFR < 60 ml/min/1.73m^2^ and/or proteinuria and/or abnormal renal ultrasound persistent for ≥ 3 months) risk factors which were noted in two studies of South African patients attending CKD clinics, were hypertension (36 to 77.8%), diabetes (25 to 29.8%) and HIV (20 to 28.5%) [30]. It is interesting to note that 51.1% of these patients had more than one CKD risk factor [34].

### Clinical and histological response to ART

In the majority of patients, renal function improved in the first year of commencing ART, as evidenced by the regression of proteinuria and the increase in eGFR [15, 31, 32]. However, there was no significant change in morphology noted on histology before and after ART initiation to explain the improvement in proteinuria [15].

## Discussion

We conducted a systematic scoping review of the available literature on the renal manifestations of HIV in the era of ART rollout in South Africa from 2004. This review provided a general overview of renal impairment (diagnostics, histological features and risk factors) in South African HIV-infected patients mainly prior to ART initiation. Most of what we know about HIV-related kidney disease has come from research performed in high income countries where the patient profiles and demographics are discordant to that of South Africa. The salient points unravelled by our review indicate a paucity of data on ART-related renal complications, specifically TDF nephrotoxicity; a deficiency of research on the impact of ART on AKI and CKD and the long-term outcomes of renal disease for patients on ART; and a relatively unknown prevalence of HIV-related kidney disease for patients on ART in South Africa. Bearing in mind that South Africa recently (1 September 2016) adopted the WHO “test and treat” approach to HIV prevention and management [3], these findings has major implications for the near future.

TDF is widely used as first line ART in South Africa since April 2010 as part of National ART guidelines [37]. A biopsy series revealed that TDF nephrotoxicity is essentially a reversible form of toxic acute tubular necrosis with features of mitochondrial injury [38]. Numerous studies have demonstrated a decrease in kidney function with TDF usage and suggest monitoring of renal function to prevent TDF nephrotoxicity [27, 39–41]. Early detection of proteinuria using accurate screening tests is important to decrease nephrotoxicity and improve outcomes in HIV-infected individuals [27]. The current South African ART guidelines recommend routinely performing urine dipstick analysis on patients before and while on ART, as well as assessing the serum creatinine and eGFR at baseline (prior to ART initiation) and then at 3 months, 6 months and annually thereafter for patients on TDF. The rate of compliance to these guidelines, however, is not known.

The use of urine dipsticks alone as a screening tool has become a contentious issue with research both locally [33] and overseas [42] suggesting the poor validity of urine dipsticks in detecting proteinuria. Data from another South African study, Han et al., suggested that HIVAN is possible in patients without overt nephrotic syndrome and in patients who have only microalbuminuria. Therefore, microalbuminuria can be considered an early marker of HIVAN [12]. Szczech et al. concluded that miroalbuminuria predicts the development of proteinuria in HIV-infected patients [43]. Stanifer et al. showed that the prevalence of proteinuria when measured in people with HIV, hypertension or diabetes is substantial. However, the best method of urine protein detection is unknown [9]. Currently, our urine dipstick analyses detect proteinuria only (not microalbuminuria). In other countries, alternate methods of urine analysis are already being sought. In a recent study set in Mexico City, a comparison was made between the protein reagent strip (PRS) and the urinary protein/creatinine ratio (uPCR) to detect proteinuria. It was concluded that there was a high concordance between the detection of urine protein by PRS and uPCR and therefore the PRS could be useful in low income countries to detect proteinuria [44].

Two South African studies in our review demonstrated the importance of an early creatinine clearance, prior to 4 months after the initiation of TDF [27, 32]. Both the CG equation (after correcting for bias) and the 4-v MDRD equation (without the ethnicity factor for African Americans) have been validated for the estimation of eGFR in Black South Africans [30]. The production of creatinine is determined mainly by muscle mass and dietary intake [45]. Therefore, using the ethnicity factor in the 4-v MDRD equation overestimates the eGFR in Black South Africans [30] due to differences in diet, muscle mass and body composition between Black South Africans and African Americans [30]. In the current review, the majority of the patients had mild to moderate renal dysfunction with a low prevalence of severe renal impairment prior to ART initiation. Similar findings were seen in a study by Overton et al. set in Washington [46]. The prevalence of significant renal impairment in a South African study [33] was slightly higher than that of a Kenyan study [47], possibly because of the differences in the two African populations and because the eGFR method used in the South African study has not been validated for use in other African countries. Though the prevalence of chronic renal failure is low in the HIV-infected population, it has been shown to be higher than that of the general population in an American study [46]. One South African study showed that approximately 6% of ART naïve outpatients who presented with proteinuria had HIV-related kidney disease [12] and another study of 99 in-patients who underwent renal biopsy showed that 27 patients (27.3%) had HIVAN, only 3 of which were on ART [17].

In the current review, HIVAN was the most common histology seen on renal biopsy [15, 31]. Other histologies have been documented in various studies both locally [12, 17, 22, 31] and overseas [48]. The third leading cause of end stage renal disease (ESRD) in African Americans aged between 20 and 64 years was found to be HIVAN [13]. HIVAN is commonly found in HIV-infected Black patients, suggesting a role of genetics in the development of HIVAN [13]. Kasembelli et al. proved that Black South Africans who carried two copies of the APOL1 risk alleles were at higher risk of developing HIVAN [28]. Lucas et al. demonstrated that ART can prevent or reduce the risk of developing HIVAN and, should it occur, patients on ART may have a slower course and lower mortality than patients not on ART [49]. Following the diagnosis of HIVAN, patients can progress to end stage renal failure within months [17]. In a South African study over a 10 year period of renal biopsies, the incidence of HIVAN increased from 6.6% in 2000 to 25.7% in 2009 [22]. Furthermore, of the 27 patients diagnosed with HIVAN on renal biopsy in a study in South Africa, only 3 were on ART [17]. This reaffirms the importance of renal biopsy in accurately diagnosing renal disease, particularly HIVAN [12, 17, 48], so that ART can be initiated early to improve outcomes. Sadly, due to a lack of resources and specialists, access to renal biopsy is limited to urban tertiary hospitals in South Africa.

From September 2016, when ART will have been made universally available to all HIV-infected South Africans in accordance with WHO ART guidelines [3], even patients with undiagnosed HIVAN will have been initiated on ART. We know from studies in other countries that renal function improves with ART. In a randomised ART trial in Uganda and Zimbabwe, there was stabilisation or a slight improvement in renal function after the initiation of ART [50]. Another study demonstrated the resolution of renal disease with ART and the recurrence of renal disease after stopping ART [51]. TDF-induced renal toxicity usually resolves after TDF cessation, but the TDF-related renal damage is not always completely reversible [52]. Our systematic scoping review demonstrated that renal function essentially improved on ART [15, 31, 32]. However, data on the long-term outcomes of renal function and CKD for patients on ART in South Africa is lacking. It was noted that 2015 had the most publications applicable to the topic of our scoping review, indicating a recent increase in interest on this subject in South Africa.

### Recommendations for future research

The majority of the included studies were conducted in an urban setting where access to healthcare and laboratories is readily available. However, research shows the high HIV prevalence and fewer patients on ART, in rural South Africa [53]. Females bear the brunt of the HIV epidemic in South Africa having a significantly higher prevalence of HIV than their male counterparts [53]. In the current review, consensus was not reached regarding gender as a risk factor for renal dysfunction possibly because females predominated in most studies. Therefore, comparative studies to determine the differences in renal manifestations between HIV-infected males and females, preferably in rural settings, are recommended. We also need to recruit more males to participate in research.

Currently, our urine dipstick analyses detect only proteinuria and not microalbuminuria. This suggests a novel idea for research: looking at the significance of microalbuminuria versus proteinuria in our HIV-infected population, the outcome of which could guide future urine diagnostics in baseline screening for renal disease. Cost-effective methods for urine screening and estimating GFR are needed, especially for rural areas where access to laboratories is limited.

Considering that the majority of South African HIV-infected patients are Black and with a genetic predisposition to HIVAN, we anticipate that the prevalence of undiagnosed HIVAN will be significant. As far as we know, there are no prospective randomised controlled trials in South Africa investigating treatment options for HIVAN and no data on the actual prevalence of HIVAN.

Data on TDF nephrotoxicity and its impact on long-term renal function are lacking in South Africa. Prospective clinical trials focusing on TDF nephrotoxicity and its long-term renal outcomes are needed.

The overall prevalence of renal disease in South Africa is unknown. We urgently need epidemiological studies assessing the prevalence of renal disease and HIV-related renal disease in South Africa in order to efficiently plan and sustain an effective CKD programme. The establishment of renal registries will assist with much needed statistics on the morbidity and mortality of renal disease in general and specifically to HIV and ART.

### Implications for practice

Risk factors associated with proteinuria and albuminuria in HIV-infected patients (low eGFR, older age, diabetes and HPT) [54, 55] overlap with those for CKD patients [56]. Stanifer et al. found that 24% of hypertensives, 18.9% of diabetics and 10% of HIV-infected patients have co-morbid CKD in Sub-Saharan Africa [9]. With the high burden of HIV and non-communicable diseases such as CKD, hypertension and diabetes in South Africa, it would be wise for the Health Department to invest in CKD clinics in rural areas and nephrology outreach services as previously proposed by Madala et al. [34]. All staff must be trained in screening for renal dysfunction and referral pathways and support systems must be in place for patients to access specialist care. In South Africa, the number of nephrologists per million population is estimated to be 1.1 [57]. The lack of specialists must be addressed in order to have optimally functioning and widely accessible CKD facilities, as well as access to renal biopsy and renal replacement services. We must build on our current infrastructure.

Lastly, we need to empower our patients with knowledge regarding ART complications, and the monitoring and recognition of side effects. In over-burdened health facilities, it is not unusual for important management steps to be overlooked. Therefore, if we educate our patients, these can be avoided.

### Strengths and limitations

An important strength of this study is the exhaustive search for relevant studies. Scoping review methodology is rigorous and methodical in its approach to examining the extent, range and nature of research activity in a particular field [18]. The unavoidable limitation of this study was the exclusion of literature published in other languages other than the English language.

## Conclusions

The findings of the review are in keeping with that of international literature. South Africa has recently adopted WHO policy and as of September 2016, all HIV-infected patients have access to ART irrespective of CD4 cell count. Though this is a victory for the millions still awaiting ART in South Africa (in this context, particularly patients with undiagnosed HIVAN), the impact on the current health infrastructure that this strategy will have is unknown. More patients on ART equates to a further increase in chronic disease in South Africa, mainly CKD, hypertension and diabetes. With a lack of dedicated CKD clinics and specialist renal services particularly in the rural settings, as well as a deficiency in knowledge on the long-term impact of ART on CKD, this may be a victory for which the healthcare system could be ill prepared.

More research is urgently needed on the impact of ART on renal disease, ART-related renal complications and the prevalence of CKD, as well as cost effective methods for routine screening of renal disease in resource-poor settings.

## Additional files

Additional file 1: Database Search Strategy. (DOCX 15 kb)

Additional file 2: PRISMA check list. (DOC 62 kb)

Additional file 3: Quality Appraisal tool. Two reviewers used the MMAT format to assess the quality of the content of the included studies. (DOCX 15 kb)

Additional file 4: Characteristics of studies included in the scoping review. (DOCX 26 kb)

## Abbreviations

ACR: Albumin creatinine ratio
AIDS: Acquired immunodeficiency syndrome
AKI: Acute kidney injury
ART: Antiretroviral treatment
CG: Cockcroft-Gault
CI: Confidence interval
CKD: Chronic kidney disease
eGFR: Estimated glomerular filtration rate
ESRD: End stage renal disease
HIV: Human immunodeficiency virus
HIVAN: HIV associated nephropathy
ICGN: Immune complex glomerulonephritis
IDSA: Infectious Diseases Society of America
MCR: Microalbumin to creatinine ratio
MDRD: Modification of Diet in Renal Disease
MMAT: Mixed methods appraisal tool
PCR: Protein creatinine ratio
PRS: Protein reagent strip
TDF: Tenofovir disoproxil fumarate
uPCR: Urinary protein creatinine ratio
WHO: World Health Organization
