# Supplementary material for: Renal manifestations of HIV during the antiretroviral era in South Africa: a systematic scoping review
Source: Syst Rev. 2017 Oct 13;6:200. doi: 10.1186/s13643-017-0605-5 (PMC5640942; doi:10.1186/s13643-017-0605-5)
Supplement: Supplementary file 1 — Database Search Strategy. (DOCX 15 kb) [file 13643_2017_605_MOESM1_ESM.docx]

**Appendix 1. Database Search Strategy**

1. **Google Scholar**

Search date: July to September 2015

Keywords: renal manifestations of HIV in South Africa

Custom range: 2004 – 2015

Number of studies found: 17300 but only able to access the first 1000 titles

Number of studies included: 89

1. **PubMed**

Search date: August 2015 to September 2015

Keywords: HIV and antiretroviral agents and renal failure or renal complications or kidney failure or proteinuria or renal tubular acidosis or glomerular filtration rate and south Africa

Custom range: 2004 – 2015

Species: Humans

Number of studies found: 259

Number of studies included: 18

1. **Cochrane Library**

Search date: August 2015 to September 2015

Keywords: renal or kidney disease or proteinuria and HIV and

antiretroviral treatment and South Africa

Custom range: 2004 – 2015

Number of studies found: 261

Number of studies included: 1

1. **WorldCat.org**

Search date: October 2015 to November 2015

Keywords: renal failure in HIV in the era of antiretroviral treatment in South

Africa

Custom range: 2004 – 2015

Number of studies found: 134

Number of studies included: 8

1. **EBSCO host**

Search date: November 2015 to December 2015

Keywords: renal failure or kidney failure and human immunodeficiency virus or HIV or proteinuria or GFR and antiretroviral therapy and South Africa.

Custom range: 2004 – 2015, English, age all adults, humans, academic journals

Number of studies found: 5090

Number of studies included: 75

1. **Medline with full text**

Search date: November to December 2015

Keywords: renal failure or kidney failure(title) and human immunodeficiency virus or HIV(title) or proteinuria(title) and south Africa(title)

Custom range: 2004 – 2015

Number of studies found: 58

Number of studies included: 22

NB. Duplicates articles removed = 41 therefore 172 records screened.
