# Supplementary material for: Renal manifestations of HIV during the antiretroviral era in South Africa: a systematic scoping review
Source: Syst Rev. 2017 Oct 13;6:200. doi: 10.1186/s13643-017-0605-5 (PMC5640942; doi:10.1186/s13643-017-0605-5)
Supplement: Supplementary file 3 — Quality Appraisal tool. Two reviewers used the MMAT format to assess the quality of the content of the included studies. (DOCX 15 kb) [file 13643_2017_605_MOESM3_ESM.docx]

**Appendix 2: Quality Appraisal Tool**

**2.1 Qualitative Studies**

| Author and date | Q1. Are there clear qualitative and quantitative research questions (or objectives*), or a clear mixed methods question (or objective*)? | Comment for Q1 | Q2. Do the collected data allow address the research question (objective)? E.g., consider whether the follow-up period is long enough for the outcome to occur (for longitudinal studies or study components). | Comment for Q2 | Q3. Are the sources of qualitative data (archives, documents, informants, observations) relevant to address the research question(objective)? | Comment for Q3 | Q4. Is the process for analyzing qualitative data relevant to address the research question (objective)? | Comment for Q4 | Q5. Is appropriate consideration given to how findings relate to the context, e.g., the setting, in which the data were collected? | Comment for Q5 | Q6. Is appropriate consideration given to how findings relate to researchers’ influence, e.g., through their interactions with participants? | Comment for Q6 | Total score | Scores presented using descriptors |
| --- | --- | --- | --- | --- | --- | --- | --- | --- | --- | --- | --- | --- | --- | --- |
| Vachiat et al. 2013 | 1 |  | 1 |  | 1 |  | 1 |  | 1 |  | 1 |  | 100% | **** |
| Fabian et al. 2013 | 1 |  | 1 |  | 1 |  | 1 |  | 1 |  | 1 |  | 100% | **** |
| Wearne et al. 2012 | 1 |  | 1 |  | 1 |  | 1 |  | 1 |  | 1 |  | 100% | **** |
| Franey et al. | 1 |  | 1 |  | 1 |  | 1 |  | 1 |  | 1 |  | 100% | **** |
| Kasembelli et al. 2015 | 1 |  | 1 |  | 1 |  | 1 |  | 1 |  | 1 |  | 100% | **** |
| Madala et al. 2014 | 1 |  | 1 |  | 1 |  | 1 |  | 1 |  | 1 |  | 100% | **** |
| Van Deventer et al. 2008 | 1 |  | 1 |  | 1 |  | 1 |  | 1 |  | 1 |  | 100% | **** |
| Fabian et al. 2009 | 1 |  | 1 |  | 1 |  | 1 |  | 0 | was not decsribed in article | 1 |  | 83% | **** |
| Wensink et al. 2015 | 1 |  | 1 |  | 1 |  | 1 |  | 0 | was not decsribed in article | 1 |  | 83% | **** |
| Brennan et al. 2011 | 1 |  | 1 |  | 1 |  | 1 |  | 1 |  | 1 |  | 100% | **** |
| Kamkuemah et al. 2015 | 1 |  | 1 |  | 1 |  | 1 |  | 1 |  | 1 |  | 100% | **** |

**2.2 Quantitative non-randomised studies**

| Author and date | Q1. Are participants (organizations) recruited in a way that minimizes selection bias? |  | Q2. Are measurements appropriate (clear origin, or validity known, or standard instrument; and absence of contamination between groups when appropriate) regarding the exposure/intervention and outcomes? | Comment for Q2 | Q3. In the groups being compared (exposed vs. non-exposed; with intervention vs. without; cases vs. controls), are the participants comparable, or do researchers take into account (control for) the difference between these groups? | Comment for Q3 | Q4. Are there complete outcome data (80% or above), and, when applicable, an acceptable response rate (60% or above), or an acceptable follow-up rate for cohort studies (depending on the duration of follow-up)? | Comment for Q4 | Total score |  |
| --- | --- | --- | --- | --- | --- | --- | --- | --- | --- | --- |
| Fabian et al. 2013 | 1 |  | 1 |  | 1 |  | 1 |  | 100% | **** |

**2.3 Quantitative Descriptive studies**

| Author and date | Q1. Is the sampling strategy relevant to address the quantitative research question (quantitative aspect of the mixed methods question)? | Comment for Q1 | Q2. Is the sample representative of the population understudy? | Comment for Q2 | Q3. Are measurements appropriate (clear origin, or validity known, or standard instrument)? | Comment for Q3 | Q4. s there an acceptable response rate (60% or above)? | Total score | Scores presented using descritors |
| --- | --- | --- | --- | --- | --- | --- | --- | --- | --- |
| Vachiat et al. 2013 | 1 |  | 1 |  | 1 |  | 1 | 100% | **** |
| Fabian et al. 2013 |  |  |  |  |  |  |  | 100% | **** |
| Wearne et al. 2012 | 1 |  | 1 |  | 1 |  | 1 | 100% | **** |
| Franey et al. | 1 |  | 1 |  | 1 |  | 1 | 100% | **** |
| Kasembelli et al. 2015 | 1 |  | 1 |  | 1 |  | 1 | 100% | **** |
| Madala et al. 2014 | 1 |  | 0 | Possible selection bias. | 1 |  | 1 | 75% | *** |
| Van Deventer et al. 2008 | 1 |  | 0 | small sample size. One site involved. | 1 |  | 1 | 75% | *** |
| Fabian et al. 2009 | 1 |  | 1 |  | 1 |  | 1 | 100% | **** |
| Wensink et al. 2015 | 1 |  | 1 |  | 1 |  | 1 | 100% | **** |
| Brennan et al. 2011 | 1 |  | 0 | small sample size. Possibility of selection bias. | 1 |  | 1 | 75% | *** |
| Kamkuemah et al. 2015 | 1 |  | 1 |  | 1 |  | 1 | 100% | **** |
