# Supplementary material for: Renal manifestations of HIV during the antiretroviral era in South Africa: a systematic scoping review
Source: Syst Rev. 2017 Oct 13;6:200. doi: 10.1186/s13643-017-0605-5 (PMC5640942; doi:10.1186/s13643-017-0605-5)
Supplement: Supplementary file 4 — Characteristics of studies included in the scoping review. (DOCX 26 kb) [file 13643_2017_605_MOESM4_ESM.docx]

**Appendix 3: Characteristics of included studies**

| **Author and date** | **Setting (rural/urban/semi-urban)** | **Sample number (n)** | **%Male** | **%Female** | **Average age** | **Intervention** | **Comparison (if applicable)** | **Outcome** | **Aim of the study** | **Study Design** | **Conclusion** |
| --- | --- | --- | --- | --- | --- | --- | --- | --- | --- | --- | --- |
| Brennan et al., 2011 | Urban | 890 | 26.5% | 73.5% | 37.1 | Either newly initiated patients on TDF or patients switched to a TDF containing regimen | ART outcome (nephrotoxicity and death) by 48-months of followup stratified by renal function at TDF initiation | Of 890 patients initiated on tenofovir, 573 (64.4%) had normal renal function (>90ml/min), 271 (30.4%) had mild renal dysfunction (60–89ml/min) and 46 (5.2%) had moderate renal dysfunction (30–59ml/min). 2.4% experienced nephrotoxicity, 7.8% died and 9.7% were lost during 48-months of follow-up. Patients with mild (HR 4.8; 95%CI: 1.5-15.2) or moderate (HR 15.0; 95%CI: 3.4-66.5) renal dysfunction were at greatest risk of nephrotoxicty, while those with mild (HR 1.2; 95%CI: 0.7-2.3) or moderate (HR 3.2; 95%CI: 1.3-7.8) renal dysfunction vs. normal renal function were at highest risk of death by 48-months. | Analyse relationship between renal dysfunction at TDF initiation, nephrotoxicity and mortality | Retrospective cohort study | All patients need to be screened for baseline renal dysfunction to decrease nephrotoxicity and improve outcomes |
| Fabian et al., 2009 | Urban | 578 | 37.5% | 62.5% | 37 | Urine dipsticks on all ART naïve, HIV infected outpatients was done | None | 84% of the screened population had AIDS (CD4 count, 200 cells/mm3), and the incidence of abnormalities onurinary dipstick testing was high: 30% had leukocyturia, 33% had microscopic hematuria, and 44% had microalbuminuria/proteinuria. In patients with leukocyturia, an infective organism was cultured in only 29.1% of cases,  predominantly Escherichia coli (70%) with sterile leukocyturia comprising the remainder. There may be an association with tuberculosis (TB) or sexually transmitted infections (STI) in the sterile leucocyturia group, but this remains to be confirmed. In those with a culture positive result the most common organism was E.Coli (70%), which exhibited 90% resistance to cotrimoxazole, demonstrating that cotrimoxazole prophylaxis is not effective to prevent urinary tract infection in this group | To detect early kidney disease in ART naïve HIV infected patients by screening for proteinuria | Cross-sectional study | Urine abnormalities in ART naïve HIV outpatients are common. Routine urine screening of all new patients attending ART clinics in South Africa should be compulsory |
| Fabian et al., 2013 | Urban | 20 renal biopsies performed out of the 578 patients screened | 55% | 45% | 37 | Kidney biopsies were performed before and after initiation of ART to assess clinical and histological response to treatment | Renal histology pre-ART compared to renal histology following initiation of ART (at variable times on ART) | There was a rapid immunological and renal response to ART. The renal response was reflected by a significant rise in the estimated glomerular filtration rate (eGFR) and rapid regression of proteinuria. The histological patterns were highly variable, ranging from non-specific lesions such as mesangial hyperplasia and interstitial nephritis to HIV-immune complex disease (HIV-ICD) with or without features of HIV-associated nephropathy (HIVAN). In the follow-up biopsies, the histological response to treatment  was variable with a combination of no change, progression or regression of lesions | To prospectively document the clinical and histological responses of biopsy proven HIV-associated renal lesions to ART | Non-randomised control study | Demonstrated a rapid virological and renal response to ART in HIV infected South Africans with histologically documented HIV associated renal disease irrespective of the histology |
| Franey et al., 2009 | Rural | 2189 in the retrospective review and 149 in the prospective study | 31.2% | 68.8% | 36 | 1.Retrospective review of medical records.  2. Single blood pressure reading, random glucose and urine analysis performed on the cohort of patients initiating ART | None | 1.Moderate renal impairment was more frequent (287/2189), low prevalence of severe renal impairment (29/2189).Age >40yrs, male gender and CD4<100 associated with significant renal impairment.  2.Urine analysis had poor sensitivity and specificity for detecting renal impairment | 1.A review of the prevalence and risk factors for renal impairment in patientss initiating ART  2.To assess the utility of urine analysis for the detection of impaired renal function in pts initiating ART | Prospective cohort study | 1.Significant renal impairment is uncommon in patients initiating ART in that rural setting. 2.Urine analysis alone may be inadequate for identification of those with impaired renal function where resources for biochemistry are limited |
| Kamkuemah et al., 2015 | Urban | 1092 | 38% | 62% | 34 | Data was from HIV-infected patients initiating TDF. Renal function was assessed for the first 12 months on ART by estimating glomerular filtration rate (eGFR) calculated using the Cockroft–Gault equation | Change in eGFR over the 12-month period | Majority had normal renal function pre-ART (79%), 19% had mildly reduced eGFR, and 2% had moderate renal impairment. Older age, more advanced WHO stage and anaemia were independently associated with prevalent renal impairment. On average, estimated glomerular function improved over the first year on tenofovir [1.10 ml/min/1.73 m2 average increase over 12 months (95% CI:0.80; 1.40)]. Male gender, anaemia and immunosuppression (WHO Stage III/IV and CD4 cell counts <100 cells/mm3) were associated with lower average eGFR levels over time. Overall, 3% developed eGFR <50 ml/min/1.73 m2 during this period. Serum creatinine tests conducted before 4 months on ART had low predictive value for predicting change in eGFR after a year on ART. | To assess the prevalence and  incidence of renal impairment in a primary care setting in sub-Saharan Africa. | Prospective cohort study | Renal function improved in HIV-infected adults initiating ART in this primary healthcare setting during the first year on ART. While monitoring of renal function is recommended in the first 4 months on ART, renal impairment appears uncommon during the first 12 months of tenofovircontaining ART in primary care populations |
| Kasembeli et al., 2015 | Urban | 228 | 42.9% HIV with CKD, 28% HIV controls, 58.1% HIV negative CKD, 44.4% population controls | 57.1% HIV with CKD, 72% HIV controls, 41.9% HIV negative controls, 55.6% populations controls | 36.1 HIV with CKD, 38.8 HIV positive controls, 36.4 HIV negative CKD, 38.5 population controls | Genomic DNA was extracted from formalin-fixed paraffin-embedded kidney samples, and a modified in house salting-out procedure was used to extract genomic DNA  from peripheral blood samples. | Comparisons were made between HIV-positive patients and HIV-positive controls and between HIV-negative patients and population controls, comparing the distribution  of two risk alleles (explanatory exposure) to zero or two risk alleles (no exposure) | 79% of patients with HIV-associated nephropathy and 2% of population controls carried two risk alleles. In a recessive model, individuals carrying any combination of two APOL1 risk alleles had 89-fold higher odds (95% confidence interval,18 to 912; P,0.001) of developing HIV-associated nephropathy compared with HIV-positive controls. Population allele frequencies were 7.3% for G1 and 11.1% for G2. APOL1 risk alleles were not significantly associated with other forms of CKD | to determine the prevalence of APOL1 risk variants and the effect of these variants on HIVAN and CKD in black South Africans in a setting of high  HIV-1 prevalence. | Case control study | HIV-positive, antiretroviral therapy–naïve South-African blacks with two APOL1 risk alleles are at very high risk for developing HIV-associated nephropathy |
| Madala et al., 2014 | Rural | 302 | 45.4 | 54.6 | 47.1 | Reviewed records, kept at the CKD clinic, of all patients seen from 31st January 2008 to 31st January 2011 | None | 290 (96%) were black African. Mean age ± SD was 47.1 ± 17.0 years. Approximately 86.4% of females and 54.5% of males were overweight/ obese. Dyslipidaemia was observed in 47.9% females and 29.2% males (P < 0.001). Estimated glomerular filtration rate (eGFR) was <30 ml/min/1.73 m2 in 50.6% patients. CKD risk factors observed were: hypertension (77.8%), diabetes (29.8%), HIV (28.5%), glomerulonephritis (7.0%) and tubulointerstitial diseases (5.6%). Independent factors associated with eGFR <30 ml/min/1.73 m2 at presentation were: HIV [OR = 2.4 (1.3-4.2), P = 0.004] and hypertension [OR = 2.3 (1.3-4.2), P = 0.007]. | To describe the prevalence of CKD and CVD risk factors and determine factors associated with CKD severity in patients presenting at a CKD clinic | Cross-sectional study | Diabetes and HIV are prevalent in CKD patients at primary/regional level healthcare in South Africa. With registry data lacking, dedicated CKD clinics at lower healthcare levels may provide valuable data on CKD epidemiology  including changes in aetiology. Primary healthcare practitioners are faced with advanced CKD patients in resource-poor settings, with limited opportunity for upward referral hence the need for nephrology outreach programs. |
| Vachiat et al., 2013 | Urban | 101 | 55% | 45% | 38 ± 9.9yrs | Review of 101 HIV-positive anti-retroviral therapy (ART)-naïve patients  presenting with renal failure from 1 October 2005 to 30 September 2006 was undertaken | HIV negative patients with acute kidney injury | Ninety-nine (98%) of HIV-positive patients were black and 56 (55%) were male, with mean age 38 ± 9.9 years (range 21–61 years). HIV-positive patients demonstrated severe immunosuppression, with mean CD4 count of 135 cells/μL (range 1–579 cells/μL). Fifty-seven (56%) HIV positive patients presented with AKI, 21 (21%) with acute-on-chronic kidney disease and 23 (23%) with CKD; seven patients with AKI were excluded due to lack of records. The causes of AKI in the HIV-positive group included sepsis (60%), volume depletion and haemodynamic instability (19%), toxins (9%), urological obstruction (7%) and miscellaneous (14%). Forty-four per cent of HIV positive and 47% of HIV-negative patients with AKI demised; P=0.45. Hyponatraemia (P = 0.018), acidosis (P=0.018), anaemia (P=0.019) and hyperphosphataemia (P=0.003) were predictors of mortality in HIV-positive patients with AKI. In comparison, predictors of mortality in the HIV-negative group were age (P = 0.023) and black ethnicity (P = 0.04). | Reviewingdata of HIV-positive patients with renal failure and primarily  compared HIV-positive and HIV-negative patients with AKI presenting in the same period, matched as closely as possible with regard to age and gender, selected as monthly consecutive referrals after the HIV-positive patients. | Retrospective cohort study | HIV-positive patients, compared with the HIV-negative group, presented with AKI at a  younger age and at an advanced stage of immunosuppression. HIV-negative patients were older and presented with more chronic comorbidities such as hypertension and diabetes. |
| Van Deventer et al., 2008 | Urban | 100 | 51% | 49% | 47 | Measurement of GFR and serum creatinine in HIV infected patients with varying degrees of renal function | Ethnicity factor established  for African Americans in the 4-v MDRD  equation. | The Spearman correlation coefficient between measured and estimated GFR for both equations was similar (4-v MDRD *R*2 _ 0.80 and CG *R*2 _0.79). Using the 4vMDRD equation with the ethnicity  factor of 1.212 as established for African Americans resulted in a median positive bias of 13.1 (95% CI 5.5 to 18.3) mL/min/1.73m2.Without the ethnicity factor, median bias was 1.9 (95% CI_0.8 to 4.5) mL/min/1.73m2 | To evaluate the performance of the 4-v MDRD and CG equations for estimating GFR in black South Africans  against measured GFR and to assess the appropriateness for the local population of the ethnicity factor established for African Americans in the 4-v MDRD equation. | Cross-sectional study | Both the 4-v MDRD equation, without the ethnicity factor of 1.212, and the Cockcroft-Gault equation, after correcting for  bias, can be used for estimating GFR in black South Africans. |
| Wearne et al., 2012 | Urban | 192 | 48.75% | 51.25% | 34 | Analysis of renal biopsies to determine outcomes and prognostic indicators based on histology and clinical features. | None | HIV-associated nephropathy (HIVAN) was the most common histology. ART reduced the mortality in those with any feature of HIVAN by 57% [adjusted hazard ratio (AHR) 0.43, 95% confidence interval (CI) 0.22–0.85]. Of those patients with HIVAN who died, 79% died of renal failure as registered on their death certificate. Proteinuria and microcysts were shown to be poor prognostic indicators (AHR 1.36: 1.09– 1.70 and 2.04: 1.24–3.37). In patients with HIVAN alone followed for up to 2 years on ART, estimated glomerular filtration rate remained stable and there was a trend towards decreased proteinuria. ART improved survival in patients with isolated immune complex disease. | Analysis of kidney biopsies to describe the histological spectrum, develop clinical correlationsand prognostic indicators and determine outcomes based on histological findings. | Cohort study | Early detection of HIV-associated renal diseases and initiation of ART in the setting of HIVAN is of paramount  importance. ART dramatically improves outcome in any patient demonstrating any of the histological features of HIVAN (with or without immune complex disease) on renal biopsy. Renal biopsy is thus essential to correctly document renal disease in the HIV-positive population. |
| Wensink et al., 2015 | Rural | 903 | 31% | 69% | 40 | HIV-infected adult patients were randomly selected and data on HIV-infection and cardiovascular risk factors were collected. Glomerular filtration rate (eGFR) was estimated | Patients who had been on ART for at least six months | The median duration since HIV diagnosis was 26 months (IQR 12–58 months) and 787 (87%) received antiretroviral therapy. Thirty-six (4%) of the subjects were shown to have diabetes and 205 (23%) hypertension. In the cohort, 21% had albuminuria and 2% an eGFR <60 mL/min/1.73m2. Albuminuria was associated withhypertension (adjusted odds ratio (aOR) 1.59;95% confidence interval (CI) 1.05–2.41; p<0.05), total cholesterol (aOR 1.31; 95% CI 1.11– 1.54; p<0.05), eGFR (aOR 0.98; 95% CI 0.97–0.99; p<0.001) and detectable viral load (aOR 2.74; 95% CI 1.56–4.79; p<0.001). Hypertension was undertreated: 78% were not receiving treatment, while another 11% were inadequately treated. No patients were receiving lipid-lowering medication. | To provide epidemiological data on the prevalence of decreased GFR and albuminuria. Additionally, to describe the association of cardiovascular and HIV-related factors with albuminuria among HIV-positive patients in a rural population in South Africa as an essential first step towards the identification of factors amenable to therapeutic intervention. | Cross-sectional study | Glomerular filtration rate was well conserved, while albuminuria was common amongst HIV infected patients in rural South Africa. Both cardiovascular and HIV-specific variables were associated with albuminuria. Improved cardiovascular risk prevention as well as adequate virus suppression might be the key to escape the vicious circle of renal failure and cardiovascular disease and improve the long-term prognosis of HIV-infected patients |

AHR = adjusted hazards ratio; AIDS = Aquired Immunodeficiency Syndrome; AKI = acute kidney injury; aOR = adjusted odds ratio; ART = antiretroviral treatment; CG = Cockcroft-Gault; CI = confidence interval; CKD = chronic kidney disease; CVD = cardiovascular disease; eGFR = estimated glomerular filtration rate; HIV = human immunodeficiency virus; HIVAN = human immunodeficiency virus associated nephropathy; HIV-ICD = human immunodeficiency virus immune complex disease; HR = hazards ratio; IQR = inter-quartile range; MDRD – Modification of Diet in Renal Disease; OR = odds ratio; SD = standard deviation; STI = sexually transmitted infection; TB = tuberculosis; TDF = tenofovir disoproxal fumarate; WHO = World Health Organization
